# Collaborative Split Learning-Based Dynamic Bandwidth Allocation for 6G-Grade TDM-PON Systems

**DOI:** 10.3390/s25144300

**Published:** 2025-07-10

**Authors:** Alaelddin F. Y. Mohammed, Yazan M. Allawi, Eman M. Moneer, Lamia O. Widaa

**Affiliations:** 1 Information Technology, Department of International Studies, Dongshin University, 67, Dongshindae-gil, Naju-si 58245, Republic of Korea; 2 Department of Electrical Engineering, College of Engineering, Princess Nourah bint Abdulrahman University, P.O. Box 84428, Riyadh 11671, Saudi Arabia; lowidaa@pnu.edu.sa; 3 Department of Physics, College of Science, Princess Nourah bint Abdulrahman University, P.O. Box 84428, Riyadh 11671, Saudi Arabia; emmohammed@pnu.edu.sa

**Keywords:** 6G, TDM-PON, DBA, split learning, machine learning, traffic prediction

## Abstract

Dynamic Bandwidth Allocation (DBA) techniques enable Time Division Multiplexing Passive Optical Network (TDM-PON) systems to efficiently manage upstream bandwidth by allowing the centralized Optical Line Terminal (OLT) to coordinate resource allocation among distributed Optical Network Units (ONUs). Conventional DBA techniques struggle to adapt to dynamic traffic conditions, resulting in suboptimal performance under varying load scenarios. This work suggests a Collaborative Split Learning-Based DBA (CSL-DBA) framework that utilizes the recently emerging Split Learning (SL) technique between the OLT and ONUs for the objective of optimizing predictive traffic adaptation and reducing communication overhead. Instead of requiring centralized learning at the OLT, the proposed approach decentralizes the process by enabling ONUs to perform local traffic analysis and transmit only model updates to the OLT. This cooperative strategy guarantees rapid responsiveness to fluctuating traffic conditions. We show by extensive simulations spanning several traffic scenarios, including low, fluctuating, and high traffic load conditions, that our proposed CSL-DBA achieves at least 99% traffic prediction accuracy, with minimal inference latency and scalable learning performance, and it reduces communication overhead by approximately 60% compared to traditional federated learning approaches, making it a strong candidate for next-generation 6G-grade TDM-PON systems.

## 1. Introduction

The exponential growth of data-intensive applications, cloud services, and connected devices has significantly increased the demand for faster, more responsive, and reliable network infrastructure. As global networks advance towards sixth-generation (6G) technology, they promise unprecedented data rates, ultra-low latency, massive connectivity, and substantial gains in reliability and efficiency [1,2]. This is specially true when considering the stringent 5G and beyond requirements for Ultra-Reliable and Low-Latency Communication (URLLC) type of applications, where delayed or failed transmission can result in severe consequences. Use cases such as disaster response, emergency medical services, and public protection increasingly depend on real-time data transmission and service orchestration over optical and wireless networks. Recent studies have declared the importance of reliability and latency guarantees in 5G and beyond for these domains, employing technologies such as network slicing, device-to-device communication, and cloud-native architectures [3,4,5,6]. These application scenarios underscore the need for intelligent, latency-aware bandwidth allocation frameworks that can adapt rapidly to varying demands and guarantee service continuity across network slices and edge infrastructures. To achieve these ambitious goals, next-generation network architectures, specifically Passive Optical Network (PON) systems, must evolve with the capability to address future demands.

Because of their high capacity, scalability, and energy efficiency, PON systems are regarded as one of the most favorable solutions for access networks [7,8]. PON technology utilizes passive splitters to minimize operational expenses (OPEX) and power consumption by eliminating the need for active electronic components in the distribution network [7,9]. Among the several types of PONs, Time Division Multiplexing-PON (TDM-PON) is a preferred option due to its simple architecture, relatively low implementation costs, and broad acceptance among optical access network Infrastructure Providers (InP) as well as Mobile Network Operators (MNO) [10,11].

The Dynamic Bandwidth Allocation (DBA) mechanism, which efficiently manages upstream bandwidth at the Optical Line Terminal (OLT) for multiple connected Optical Network Units (ONUs), is a core component of TDM-PON architecture. By dynamically allocating bandwidth based on real-time demand, traffic patterns, and Quality of Service (QoS) requirements, DBA significantly enhances overall network performance [7,12]. However, with the dynamic and diverse network environments anticipated for 6G-grade services and applications, conventional DBA approaches exhibit significant limitations that may hinder their effectiveness. Moreover, existing standards (e.g., IEEE 1904.1 [13] and ITU-T G.988 [14]) intentionally leave room for innovation, encouraging manufacturers and researchers to explore enhanced DBA designs.

Conventional DBA systems face several challenges, particularly in terms of latency, due to their reliance on centralized bandwidth allocation decisions at the OLT. This centralized approach requires continuous and frequent communication between ONUs and the OLT, which exacerbates latency, especially under heavy network load or rapidly changing traffic conditions [15,16]. Additionally, traditional DBA mechanisms often struggle to respond effectively to sudden shifts in traffic demand, resulting in inefficient bandwidth distribution and compromised QoS. A further concern lies in privacy and security risks, as centralized data processing at the OLT can expose sensitive user information, which represents a significant threat in mission-critical and high-security applications [17,18].

Recent advancements in response to these challenges have proposed enhancing DBA mechanisms by integrating Machine Learning (ML) and Deep Learning (DL) approaches. ML-based solutions enable predictive analytics to dynamically allocate bandwidth in real time, improving traffic prediction accuracy [15,19]. However, these systems often rely on centralized architectures, which aggravate privacy concerns and introduce significant computational and communication overhead. This overhead limits their practical applicability, particularly as networks scale to meet 6G-grade QoS demands [20], while existing ML-based DBA systems have demonstrated progress, they remain inadequate for addressing the distinct challenges of 6G networks, including ultra-high bandwidth requirements, extremely low latency demands, and the necessity for flexible and real-time adaptation to heterogeneous traffic environments [10,21]. To overcome these limitations, the emerging Split Learning (SL) technique, recently introduced by MIT’s Media Lab [22,23], presents a promising solution. As a variation of Collaborative Learning (CL), SL enables multiple parties to collaboratively train different neural network segments by exchanging only intermediate features, thus preserving raw data privacy. Given its inherent characteristics of decentralized processing, scalability, and privacy preservation, an SL-based approach can be particularly advantageous for next-generation TDM-PON environments. While traditional ML and Federated Learning (FL) approaches have shown potential in DBA, they present significant limitations. ML typically relies on centralized training, which increases communication overhead and introduces privacy risks, especially under high ONU density. FL partially addresses privacy by decentralizing model training, but it still requires full model parameter exchange between clients and the central node, resulting in large communication loads and synchronization issues. On the other hand, SL, as implemented in our proposed framework, only exchanges partial forward and backward features, significantly reducing communication cost while preserving data privacy.

In this paper, we introduce a novel Collaborative Split Learning-based Dynamic Bandwidth Allocation (CSL-DBA) technique tailor-designed for next-generation 6G-grade TDM-PON systems. Our approach decentralizes traffic prediction and bandwidth allocation through the application of SL, to distribute learning tasks between ONUs and the OLT, thus facilitating efficient knowledge sharing while preserving user privacy. Unlike traditional centralized DBA techniques, the CSL-DBA approach significantly reduces the latency caused by frequent round-trip signaling in the OLT-ONU-OLT communication cycles. This is made possible under the proposed architecture by enabling the ONUs to independently estimate local bandwidth requirements using lightweight models built from historical and real-time traffic data. Subsequently, ONUs only need to transmit model gradients to the OLT, without including any real user data, which guarantees the preservation of user privacy. The OLT then combines these gradients by means of the proposed cooperative approach to enhance prediction accuracy, as well as achieving lower latency and higher adaptability to the rapidly fluctuating traffic demands expected in 6G-grade TDM-PON systems.

To the best of the authors’ knowledge, this work is the first to explore the potential advantages of utilizing SL for enhancing the performance of DBA in TDM-PON systems and to propose a collaborative split learning-based DBA framework specifically tailored to the requirements and characteristics of 6G-grade TDM-PON systems. The results presented in this paper offer a foundational reference for optimizing bandwidth allocation in the context of next-generation optical communication networks, highlighting the significant potential of the proposed approach. The remaining part of this paper is organized as follows: Section 2 reviews the state-of-the-art DBA techniques and the integration of ML-based and SL-based approaches in the context of 6G networks. In Section 3, we explain our proposed CSL-DBA architecture together with its system model and design principles. Simulation results are presented and discussed in Section 4, showcasing the efficiency of our proposed design. Section 5 summarizes the main contributions presented in this work and identifies future research directions.

## 2. Related Work

Controlling the upstream bandwidth allocation among connected ONUs in TDM-PON systems depends on the DBA. Traditional DBA techniques, such as Interleaved Polling with Adaptive Cycle Time (IPACT), remain popular due to their simplicity. However, these approaches exhibit significant limitations in latency, scalability, and responsiveness when considered for handling the dynamic environments anticipated for 6G optical networks [9,11,15,24]. Recent advancements in DBA include the optimization of adaptive traffic management and resource efficiency by implementing a QoS-aware DBA algorithm that accounts for adjusting times in PON systems to improve bandwidth usage and latency performance [21].

The promise of more accurate traffic demand predictions for improved DBA management efficiency has driven high interest in ML-based approaches. Focusing on possible improvements in proactive resource management. Authors in [25] presented a thorough study of the potential applications for ML-based models in optical networks. In addition, different DL-based DBA approaches for estimating bandwidth demands in the context of XG-PON intended for mobile fronthaul within Cloud Radio Access Networks (C-RANs) were introduced in [19]. By means of simulations, the authors demonstrated that DL-based DBA approaches results in noticeable improvement in system efficiency and performance over conventional DBA techniques, and further suggested that DL-based models, if not designed properly, can increase CPU consumption, which can potentially lead to performance degradation in highly resource-limited environments.

Furthermore, a Deep Neural Network (DNN) approach for traffic prediction in PONs was proposed in [15], aiming at enhancing bandwidth allocation efficiency. The work in [24] presented a Deep Reinforcement Learning (DRL) method for network slicing in heterogeneous and dynamic traffic environments. Their approach seeks to improve resource allocation by means of adapting to varying traffic conditions and their QoS requirements. The system constantly learns and improves its allocation strategies using DRL. Particularly in multi-tenant network architectures, the study shows efficient bandwidth allocation and improved resource usage. Although their approach is effective in improving responsiveness to dynamic traffic conditions, it does not particularly address the special requirements and settings of TDM-PON systems. Despite these advancements, the core issue lies in the centralization of all tasks related to traffic monitoring, prediction, and resource allocation at the OLT, which limits scalability and raises privacy concerns.

Recent research endeavors have been focusing on the privacy and scalability issues present in existing DBA approaches. To help reduce vulnerabilities in next-generation PON systems, the authors in [26] proposed a security-enhanced DBA algorithm, which introduces a mitigating phase that reduces bandwidth allocation when identifying attackers and another detection phase for spotting unusual behavior. The work in [27] offers an attack-aware DBA technique for PON for addressing security flaws in traditional DBA techniques, which comprises an intelligent detection and allocation strategy to guarantee fair bandwidth distribution, even in hostile environments, by means of dynamic traffic monitoring and anomaly detection for effective resource utilization and enhanced privacy within the network. Nevertheless, existing detection techniques fall short when addressing highly-fluctuating traffic conditions or when dealing with large-scale networks.

In view of the aforementioned challenges, this paper proposes a collaborative, semi-supervised, split-learning (CSL)-based approach, referred to as CSL-DBA, specifically designed for next-generation 6G-grade TDM-PON systems. Our architecture delegates traffic prediction tasks to the ONUs, thereby significantly reducing latency, preserving user privacy, and enabling real-time adaptability to diverse network conditions, as detailed in the following section.

## 3. Network Architecture and Proposed System Model

### 3.1. TDM-PON System Architecture

**Figure 1:**
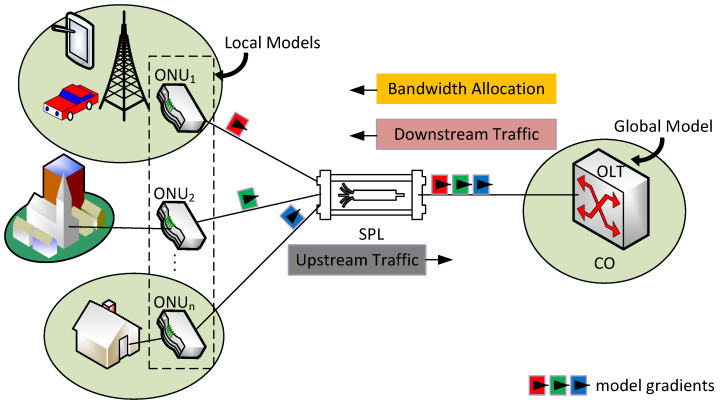
TDM-PON system architecture with CSL-DBA-based bandwidth allocation.

Figure 1 shows a detailed representation of the proposed TDM-PON system architecture with CSL-DBA-based bandwidth allocation. The figure illustrates how multiple ONUs independently analyze local traffic and collaboratively train the global model at the OLT, which supports scalable bandwidth prediction and allocation in real-time multi-user environments. The standard optical access network topology consists of a number of ONUs at the user locations, a passive optical splitter (SPL), and an OLT at the Central Office (CO). This design sends downstream data traffic from the OLT over broadcast transmissions to all ONUs. On the other hand, upstream traffic from ONUs pass via the SPL to the OLT. Generally, in TDM-PON systems, eliminating collisions and guaranteeing optimal network performance solely depends on the DBA, which is responsible for the allocation and synchronization of the available bandwidth as well as the scheduling of transmissions for all ONUs that use a single optical fiber link in both directions.

### 3.2. Proposed CSL-DBA System Model

We propose a hybrid model that integrates SL-based approach and a DBA system, whereby the learning task is split between the distributed ONUs and the centralized OLT. The proposed CSL-DBA system assumes the role of distributing learning tasks among the ONUs and coordinating the exchange of prediction-related information between the OLT and ONUs. By utilizing SL, ONUs localize computations and traffic pattern analysis to enable capturing real-time bandwidth requirements and network-edge variations. As a result, instead of providing raw data or comprehensive traffic statistics, ONUs send only their locally generated model updates (i.e., gradient information) to the centralized OLT. After receiving these updates, the OLT compiles them to improve and preserve the global predictive model, which is then used to dynamically and actively allocate bandwidth. Furthermore, as unprocessed traffic data are excluded from crossing the whole network segment for centralized processing, the proposed CSL-DBA significantly reduces computing load, delay, and communication overhead.

Figure 1 also shows a conventional PON arrangement and points out particular integration sites for CSL-DBA. At the ONUs, which are located at the network edge, local predictive learning is carried out such that the OLT in the network core handles global model aggregation and resource allocation decisions. SL-based models are trained by the ONU and OLT to dynamically maximize bandwidth allocation. Our system comprises a lightweight SL-based local models at the ONU side, which serve the purpose of forecasting local bandwidth needs that are captured as follows:

(1) $$B_{D}^{\mathrm{ONU}}\left(t\right)=\phi_{L}\left(X_{t-1}^{\mathrm{ONU}},X_{t-2}^{\mathrm{ONU}},\ldots ,X_{t-n}^{\mathrm{ONU}}\right)+\epsilon_{L}\left(t\right),$$

where $\phi_{L}$ represents the local predictive model; $X_{t-i}^{\mathrm{ONU}}$ encapsulates features such as historical bandwidth usage, queue lengths, and latency metrics; and $\epsilon_{L}\left(t\right)$ accounts for the prediction uncertainty of local traffic.

Furthermore, ONU assists in predicting different traffic patterns (i.e., voice, video, and data) in DBA for TDM-PON systems as expressed in the following equation:

(2) $$P_{F}\left(t\right)=\phi_{LF}\left(T_{t-1}^{\mathrm{ONU}},T_{t-2}^{\mathrm{ONU}},\ldots ,T_{t-n}^{\mathrm{ONU}}\right)+\epsilon_{L}\left(t\right),$$

where $P_{F}\left(t\right)$ is the predicted traffic (*F*) pattern at time *t*, $\phi_{LF}$ is the model predicting local traffic type and patterns, and $T_{t-1}^{\mathrm{ONU}},T_{t-2}^{\mathrm{ONU}},\ldots ,T_{t-n}^{\mathrm{ONU}}$ are the past traffic types and patterns observed.

Traffic prediction allows the OLT to dynamically change time slot assignments depending on expected demand, enabling adaptive DBA scheduling. By guaranteeing fair resource distribution and lowering contention among ONUs, this increases network efficiency while following Service-Level Agreements (SLAs). In practical deployment scenarios, the behavior of users in different ONUs is highly unpredictable and not easily controllable. These variations can cause inconsistent and bursty traffic demand at multiple stations. The proposed CSL-DBA addresses this challenge through its distributed learning approach, where each ONU independently observes and learns from its localized traffic characteristics (see Figure 1). These local models were trained on historical and real-time traffic patterns, which allow the system to dynamically anticipate bandwidth needs without relying on static assumptions. In ONUs experiencing limited bandwidth, the global model at the OLT adjusts time slot assignments in real time by prioritizing ONUs with higher predicted demands or QoS sensitivity. This allows for fairness and responsiveness even under constrained network conditions. The collaborative structure confirms the system remains scalable and adaptive to real-life user behavior diversity.

Upon local training, each ONU, denoted by *i*, transmits its model gradient $\nabla L_{L,i}$ to the OLT. The OLT then aggregates all received gradients using an equal-weighted average to compute the global update. This aggregation strategy follows a Federated Averaging (FedAvg)-like approach and guarantees that each ONU contributes equally to the updated model $\theta_{G}$ as follows:

(3) $$\theta_{G}^{\prime }=\theta_{G}-\eta \cdot \frac{1}{N}\sum_{i=1}^{N}\nabla LL,i+\lambda R\left(\theta G\right),$$

where $\theta_{G}^{\prime }$ is the updated global model, η is the learning rate, *N* is the number of participating ONUs, λ is a regularization parameter, and $R(\theta_{G})$ is a regularization function (e.g., L2 penalty) used to improve model generalization and prevent overfitting [20]. After updating the local model at each ONU, all gradients of local models are aggregated and processed at OLT’s global model to predict optimal bandwidth allocation using an optimization-based approach, as expressed in (4),

(4) $$B_{alloc}^{\mathrm{OLT}}=\arg\max_{B^{pred}}\left[\sum_{i=1}^{N}U\left(B_{i,t}^{pred}\right)-\mu \sum_{i=1}^{N}C_{i}\left(t\right)\right],$$

the global model, updated via aggregated gradients from ONUs, learns mapping from ONU-level traffic features to predicted bandwidth demand. These predictions, denoted as $B_{i,t}^{pred}$ for each ${\mathrm{ONU}}_{i}$, serve as inputs to the OLT’s bandwidth allocation decision. Specifically, the OLT uses these predictions according to the optimization model in (4) that maximizes utility $U(B_{i})$ while satisfying QoS constraints $C_{i}\left(t\right)$. Thus, the optimization in (4) is driven by predicted demands. To further enhance the efficiency of bandwidth allocation, a second stage for allocation correction is performed based on (5), which serves as a feedback-based correction that adjusts the allocation using real-time queue length deviations, ensuring responsiveness to transient congestion.

(5) $$B_{alloc}^{final}=B_{\mathrm{OLT}}^{alloc}+\alpha \sum_{i=1}^{N}\left(Q_{i}\left(t\right)-\bar{Q}\right),$$

where $Q_{i}\left(t\right)$ is the queue length of ${\mathrm{ONU}}_{i}$, $\bar{Q}$ is the mean queue length, and α is an adaptive correction coefficient.

**Figure 2:**
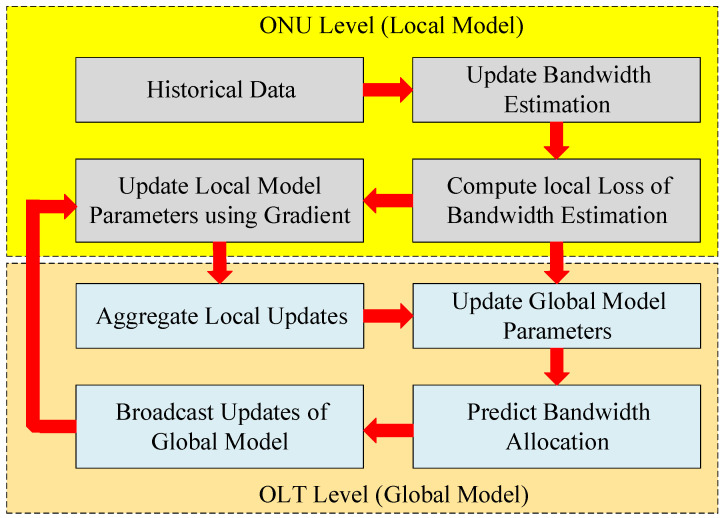
Flow diagram of the proposed CSL-DBA framework. The system operates across the following two levels: the ONU level, where local demand is predicted and gradients are computed based on historical traffic data; and the OLT level, where gradients are aggregated and the global model is updated and broadcast back to ONUs for subsequent use in local predictions.

The framework of our proposed model is shown in Figure 2, which illustrates the two primary levels of the overall process as follows: the local model (ONU level) and the global model (OLT level). Starting at the ONU level, each ONU relies on historical patterns of bandwidth usage, queue lengths, and latency measurements to guide its current activities. By means of local traffic patterns, this data aid in future bandwidth demand predictions. After that, the expected demand moves to a loss computation module, which is a process guided by predictive accuracy. This loss drives the adaptation of local model parameters. Each ONU sends model update gradients or partial weights to the OLT, instead of unprocessed traffic data, once the local training phase ends. These local model gradients reflect the acquired learning from the observed ONU traffic conditions. Each ONU individually sends its local updates, which are then gathered and processed by the global model at the OLT Level. This approach results in regularly updating the global model parameters, considering traffic patterns, all over the network from an integrated point of view. In the next scheduling cycle, ONUs can forecast their traffic patterns using the updated local models.

### 3.3. CSL-DBA Training and Update Cycle

Algorithm 1 summarizes the key steps involved in the model update cycle between the ONUs and the OLT, which provides a clearer understanding of the collaborative training workflow in CSL-DBA. Each ONU observes local traffic features, performs lightweight forward inference and local training using its MLP-based predictor, and computes gradients of the loss function based on predicted vs. actual bandwidth demands. These local gradients are transmitted to the OLT, which aggregates them to update the global model. The updated model parameters are then broadcast back to the ONUs to complete the cycle. This process is repeated across multiple rounds, enabling the global model to evolve based on distributed real-time observations while maintaining user data privacy.
**Algorithm 1** Training and update cycle.  1:**Initialize:** global model $\theta_{G}$, local models $\theta_{L,i}$ for each ONU_*i*_, learning rate η, update frequency $f_{u}$  2:**for** each global training round t=1 to *T* **do**  3:      **for** each ONU_*i*_ in parallel **do**  4:            Local traffic features observation: $X_{i,t-n:t-1}$  5:            Local bandwidth prediction: $B_{i,t}^{pred}=\phi_{L}\left(X_{i}\right)$  6:            Local loss computation: $L_{i}=L\left(B_{i,t}^{pred},B_{i,t}^{true}\right)$  7:            Local gradient computation: $\nabla L_{i}$  8:            Send $\nabla L_{i}$ to OLT  9:      **end for**10:      Gradients aggregation at the OLT: $\nabla L_{G}=\frac{1}{N}\sum_{i=1}^{N}\nabla L_{L,i}$$L_{i}$11:      Update global model: $\theta_{G}\leftarrow \theta_{G}-\eta \cdot \nabla L_{G}$12:      Broadcast global model to ONUs13:**end for**

In our implementation, the local model at each ONU is a lightweight Multilayer Perceptron (MLP) consisting of an input layer, two hidden layers with 64 and 32 neurons, respectively, and a ReLU activation function. The model takes as input a feature vector of 10 traffic-related attributes (e.g., historical queue lengths, latency, and bandwidth demand), and it produces a single output predicting the required bandwidth in (1) or traffic class in (2). This MLP architecture was selected due to its low computational cost and rapid convergence, making it well-suited for deployment in resource-constrained ONUs. Moreover, MLPs offer a good trade-off between accuracy and latency for time-series forecasting in fixed-length input formats. While more complex models like LSTM or CNN may offer marginal improvements in accuracy, they significantly increase inference latency and training overhead, which may not be acceptable in real-time PON environments. To support SL, the model is partitioned such that the input and first hidden layer (64 neurons) are hosted locally at each ONU, while the second hidden layer (32 neurons) and output layer are executed at the OLT. This division allows ONUs to perform lightweight local computations and transmit only intermediate activations, while the OLT completes the forward/backward pass and updates the global model based on aggregated gradients.

It has to be noted here that the lightweight SL-based local models at the ONUs are not separate early-exit variants but rather the front portion of a globally shared MLP model partitioned across the network. Specifically, each ONU hosts the input and first hidden layer, while the remaining layers reside at the OLT. During inference, the ONU performs the forward pass through its portion of the model and transmits the resulting intermediate activation (feature vector) to the OLT. The OLT then completes the forward pass through the remaining layers to generate the final prediction. This behavior is consistent with the training process in Split Learning and avoids redundant computation while preserving model consistency.

## 4. Results and Discussion

We created a large-scale simulation environment, using the TensorFlow (2.18.0) tool [28], in order to conduct a comprehensive performance evaluation for our proposed CSL-DBA approach. The commonly used open source ML toolkit made available by TensorFlow provides complete support for developing and training sophisticated SL-based models. The distributed learning interactions between the OLT and the ONUs are faithfully depicted, which allows us to replicate several traffic patterns required in various operational settings. The performance of our proposed CSL-DBA, in terms of its effectiveness and usability, is demonstrated through extensive simulations to capture the complexity and dynamicity of various TDM-PON systems mimicking real-world scenarios.

To evaluate CSL-DBA under varying network conditions, we modeled three common traffic scenarios using Poisson arrival processes, as follows:

- **Low traffic:** Poisson arrivals with mean rate λ=0.2 packets/slot per ONU, representing under-utilization.
- **High traffic:** Poisson arrivals with λ=0.8 packets/slot per ONU, simulating near-saturation conditions.
- **Fluctuating traffic:** Time-varying Poisson rates alternating between λ=0.2 and λ=0.8 every 1000 slots, to mimic bursty, real-world traffic dynamics.

These parameters align with typical values used in recent DBA and TDM-PON studies in [29]. Our synthetic dataset includes up to 10,000 time slots of simulated traffic traces per scenario, encompassing queue length, delay, and arrival rate statistics.

### 4.1. Training and Inference Latency

This section offers a comparison of the Cumulative Distribution Function (CDF) for training and inference latency over the following three separate traffic scenarios: low, fluctuating, and high traffic with ONU counts of 8, 32, and 64, in each scenario. This will allow us to understand how delay behaves as network complexity and traffic conditions vary.

The training latency shown in the CDFs represents the time taken to complete one epoch of local training at each ONU, averaged across the simulation window. Each epoch includes a full pass over the local traffic samples for that round. Inference latency, on the other hand, corresponds to the time required to process a single input feature vector (i.e., one traffic sample) during prediction. This latency includes both forward propagation at the ONU (for the local model segment) and the continuation of inference at the OLT (for the server-side model segment), as dictated by the Split Learning structure.

**Figure 3:**
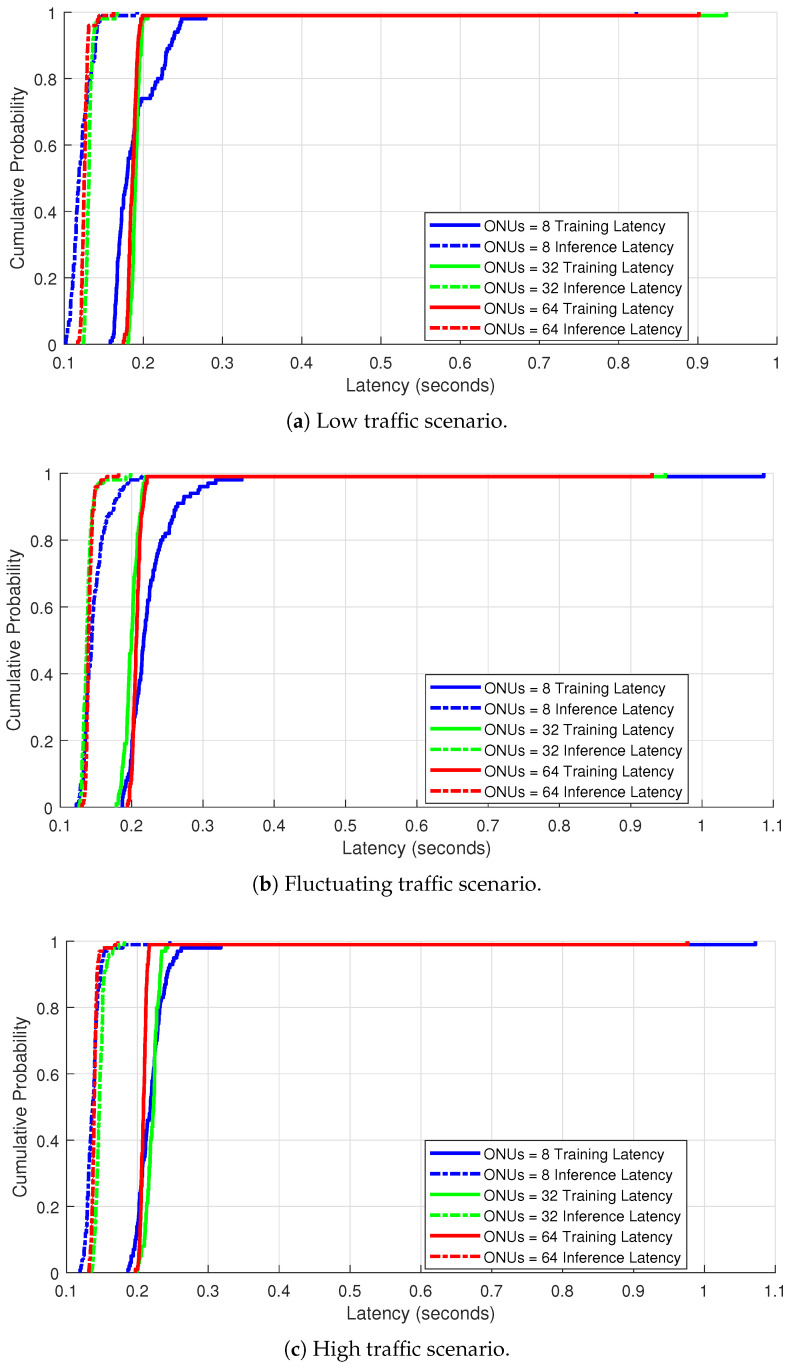
Training and inference latency distributions under different traffic scenarios.

Under the low traffic scenario, as illustrated in Figure 3a, for all ONU configurations, training and inference latencies are tightly coupled below 0.2 s. The system allows 8 ONUs with low latency, implying that a smaller number of ONUs speed up training and inference processes because of less data contention. We also notice that there is a slight increase in latency occurring when the number of ONUs rise to 32 and to 64, which indicates that the collaborative learning model can efficiently scale under smaller loads. Nevertheless, the difference in training and inference delay time is still negligible.

On the other had, under fluctuating traffic conditions, as presented in Figure 3b, we observe that our proposed CSL-DBA model adapts well to mid-level unpredictable traffic, while training latency continues to be manageable even as the number of ONUs scales up. At the same time, the inference latency remains tightly bounded, showing robustness in generating real-time inference.

Figure 3c shows that training becomes more resource-intensive with the size of the network. However, the CSL-DBA model is still capable of handling inference very well, with a slight increase in latency spread. This indicates that our CSL-DBA approach generalizes effectively, supporting training under low, static, and congested traffic conditions. This capability is particularly advantageous, given that training latency and fairness have been identified as critical bottlenecks hindering the effectiveness of TDM-PON’s DBAs under high traffic loads and increasing numbers of ONUs, as highlighted in [15,17].

### 4.2. Training Versus Validation Loss

**Figure 4:**
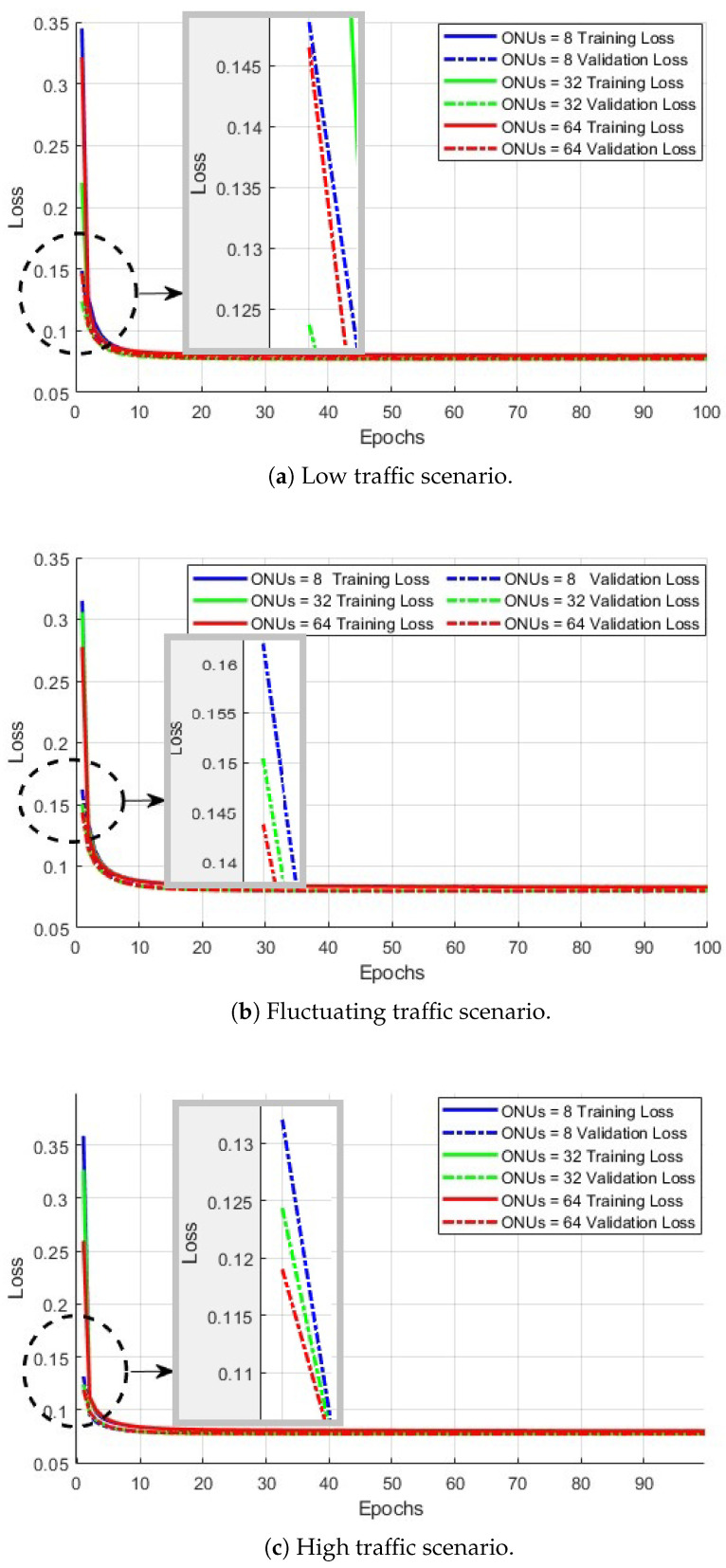
Training versus validation loss under different traffic conditions for 8, 32, and 64 ONUs.

The relationship between training and validation losses provides insights into potential performance issues such as overfitting, underfitting, slow convergence, or instabilities during model training. Figure 4a illustrates the low traffic scenario, where both training and validation losses exhibit a steady gradual decline. The close alignment of the two curves indicates the absence of overfitting and suggests strong generalization to unseen data. The rapid convergence to a minimal loss demonstrates the model’s ability to enhance the predictability of bandwidth demands, which enables efficient resource utilization with minimal performance degradation. In such a low traffic scenario, the availability of high-quality training data and the absence of significant demand fluctuations contribute to an optimal learning environment for our proposed model.

Figure 4b presents the training and validation loss curves for the fluctuating traffic scenario. Similar to the previously described behavior of low traffic scenario, both training and validation losses decrease smoothly and consistently across all configurations, with no significant spikes or instability observed. While validation loss varies only during the first three epochs, the proposed model continues learning and does not collapse, demonstrating robustness and the ability to track rapid changes in bandwidth demands. Furthermore, we see that the convergence behavior is similar across the different ONU counts (8, 32, and 64), with loss values stabilizing at a marginal value of 0.08. The close alignment between training and validation curves suggests that the model maintains strong generalization capabilities, even under fluctuating traffic conditions, indicating reliable and stable learning dynamics.

Figure 4c presents the training and validation loss curves under a high traffic scenario, and the results show that the overall trend remains stable with minimal overfitting across all configurations. The consistent convergence for both training and validation losses demonstrates the model’s scalability and robustness in handling high traffic loads and effective adaptability to the complexity and dynamicity of the traffic patterns encountered in high-load scenarios without significant degradation in performance. On top of the low and converging validation losses, there is no evidence of overfitting or instability. Therefore, while further enhancements such as regularization techniques (e.g., weight decay or dropout [30]) might offer marginal gains, the current training configuration already yields strong performance under high traffic conditions.

### 4.3. Accuracy and Inference Latency

In time-sensitive network environments such as TDM-PON, maintaining high inference accuracy while minimizing latency is critical for ensuring responsive and efficient bandwidth allocation. In this subsection, we explore the interplay between prediction accuracy and inference latency under different traffic conditions, providing insights into the suitability of our proposed CSL-DBA model for real-time deployment. By evaluating how CSL-DBA performs across different traffic conditions and ONU configurations, we assess its ability to scale while sustaining precise predictions with minimal delay.

**Figure 5:**
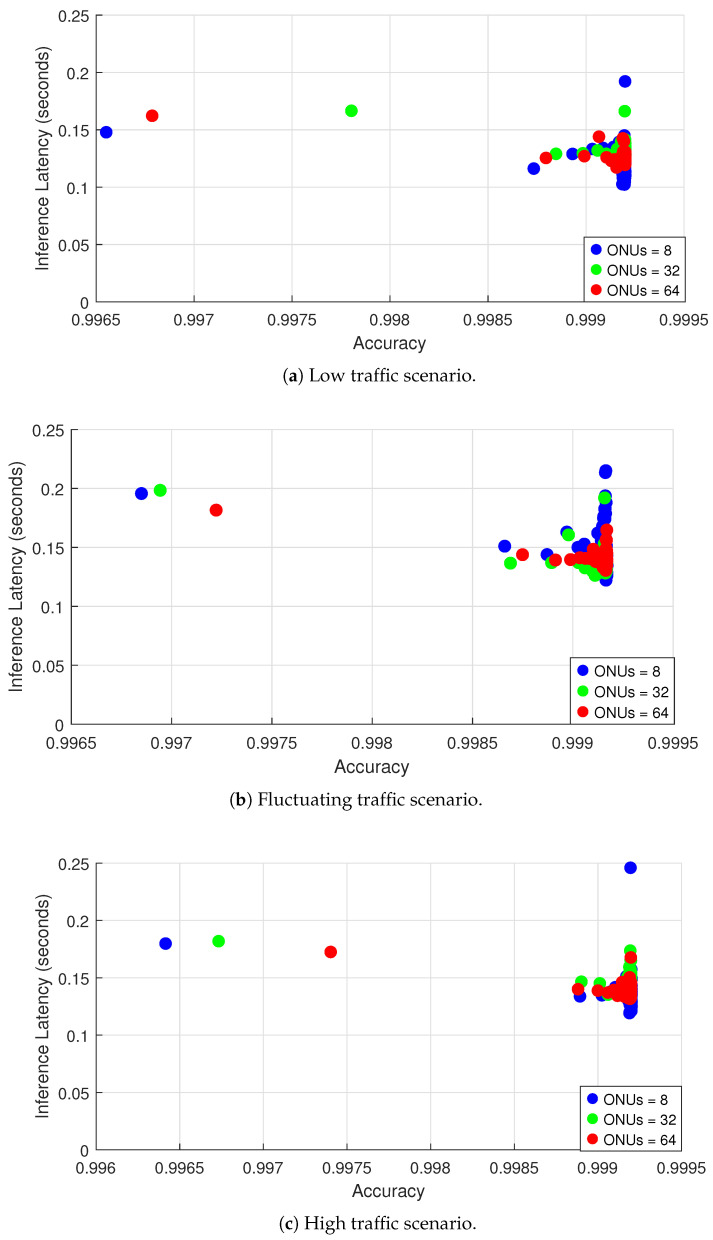
Accuracy versus inference latency under different traffic conditions for 8, 32, and 64 ONUs.

Figure 5a illustrates the performance of the proposed model in a low traffic scenario, were the results indicate that our CSL-DBA model mainaining high accuracy of above 99.6%, while maintaining low inference latency across all ONU configurations. This demonstrates the model’s capability to deliver reliable bandwidth predictions with minimal computational delay when operating under lightly loaded network conditions. The observed distribution of data points shows that increasing the number of ONUs, from 8 to 32 and to 64, has a negligible impact on both accuracy and latency. Inference latency remains consistently below 0.1 s, indicating that the collaborative learning process introduces minimal overhead. Thus, the proposed model continues to perform optimally even as the network grows in size.

Figure 5b presents the model’s performance under fluctuating traffic conditions. Here, we notice that the accuracy remains high across all configurations, consistently exceeding 99.7%, although slight variations are observed in the 32 and 64 ONU setups. As the traffic becomes more dynamic and unpredictable, the increased complexity introduces a slightly higher computational demand, met by a rise in inference latency, especially in the case of 64 ONUs, where latency reaches up to 0.15 s. Such a modest drop in accuracy compared to the low traffic scenario suggests that the model is capable of facing the challenge of adapting to rapid and irregular fluctuations in bandwidth demand. While this is expected, as such volatility, which introduces an uncertainty that complicates the generalization of learned patterns, the model maintains robust performance, demonstrating resilience and adaptability with an acceptable trade-off in latency under high traffic variability conditions.

Finally, Figure 5c, shows the results in the case of a high traffic scenario, While the accuracy of our proposed CSL-DBA model remains consistently high with a score above 99.6%, there is a noticeable increase in inference latency corresponding to the larger network size, where it approaches 0.25 s for the case of 64 ONUs. This reflects a higher processing burden associated with managing large-scale networks under heavy congestion. Factors such as queuing delays and high variability in bandwidth demand usually intensify computational complexity; however, our CSL-DBA model successfully maintains real-time performance with a stable and high accuracy, underscoring its robustness in congested environments. However, the findings also highlight a scalability challenge; as the number of ONUs and traffic intensity increase, the cost in inference time becomes more pronounced.

Hence, the proposed CSL-DBA model demonstrates strong resilience across all traffic conditions, reliably forecasting bandwidth allocation. Yet, the observed growth in inference latency under high traffic conditions emphasizes the need for continued refinement to guarantee scalability and responsiveness in next-generation TDM-PON systems. To preserve real-time adaptability in large-scale deployments, future enhancements should focus on reducing computational overhead—potentially through model simplification or latency-aware optimization techniques [31,32].

### 4.4. Communication Overhead Analysis

An important metric for evaluating the scalability of learning-based DBA approaches is communication overhead, which refers to the volume of data exchanged between ONUs and the OLT during model updates. Traditional ML-based DBA requires centralized data aggregation at the OLT, leading to high upstream bandwidth usage and significant latency. FL-based DBA frameworks partially address this by decentralizing model training; however, they still involve full model weight exchanges in every training round, which may be impractical for resource-constrained ONUs.

To quantify the overhead, we implemented a baseline FL-based DBA model using the same local prediction model at each ONU as used in CSL-DBA, trained under a standard FL protocol (i.e., local training followed by global aggregation). Both approaches used identical layer structures and optimizer configurations to guarantee a fair comparison. The critical difference lies in the data exchange; FL requires each ONU to send the full model weights (typically 50–100 KB) to the OLT per training round, resulting in an average uplink usage of approximately 200 KB per ONU.

On the other hand, CSL-DBA significantly minimizes communication overhead by transmitting only intermediate activations and partial gradients. Based on simulation logs and profiling using TensorFlow, the average uplink size per ONU was measured at approximately 80 KB. This results in a communication overhead reduction of roughly 60% compared to the FL baseline. When compared to centralized ML approaches that require raw traffic data transmission from each ONU, the overhead reduction can exceed 80%.

### 4.5. Benchmarking CSL-DBA with Existing Solutions

The proposed CSL-DBA framework introduces a novel approach to bandwidth allocation in next-generation 6G-grade TDM-PON networks. To highlight its advantages, we systematically compare its functional capabilities with those of existing DBA schemes reported in the literature. Table 1 presents a comparative analysis between the CSL-DBA framework and various state-of-the-art DBA approaches. The comparison clearly demonstrate that CSL-DBA outperforms existing solutions in several key areas, particularly in traffic adaptability and prediction accuracy under dynamic load conditions. Unlike most conventional and ML-based DBA models that rely on centralized control at the OLT and are typically tuned for average or stationary traffic, such as those discussed in [15,19], our proposed CSL-DBA approach benefits from its utilization of the SL model to enable decentralized, real-time decision making without compromising efficiency or scalability.

Rule-based and QoS-oriented models [11,21] exhibit limited adaptability when confronted with bursty or non-uniform traffic, often resulting in reduced responsiveness. Similarly, security-focused DBA methods [26,27] typically overlook intelligent resource forecasting and lack general applicability across diverse network conditions. In contrast, the proposed CSL-DBA is capable of achieving an accuracy above 99.6% in variable and high traffic scenarios due to its collaborative learning mechanism, which effectively captures complex traffic dynamics between ONUs and the OLT.

Although recent architectural enhancements, such as fronthaul-aware and Mobile Edge Computing (MEC)-integrated DBA frameworks [1,8], provide improved infrastructure integration, they still lack an intelligent, adaptive learning layer. In contrast, CSL-DBA addresses this gap by offering a scalable, learning-driven solution optimized for next-generation optical networks. This confirms that the proposed CSL-DBA is a highly flexible, intelligent, and 6G-ready bandwidth management framework that meets the performance, privacy, and responsiveness demands of next-generation TDM-PON systems.

Compared to Federated Learning (FL), CSL-DBA offers improved communication efficiency and better suitability for asymmetric, low-power ONUs. While FL requires the full exchange of model weights after each training round, SL only transmits intermediate representations, making it lightweight and less demanding for ONU hardware. Moreover, SL inherently decouples forward and backward passes between ONUs and the OLT, allowing asynchronous execution and better latency control. These characteristics make CSL-DBA better suited for real-time DBA tasks in dynamic and large-scale TDM-PON environments.

Our proposed CSL-DBA framework is unique for TDM-PON networks, thus we systematically compare its functional characteristics with those of current advanced DBA systems published in the literature. Table 1 shows a comparison of our suggested CSL-DBA framework with current DBA approaches. The results unequivocally show that CSL-DBA outperforms present approaches in many important respects, especially with relation to traffic flexibility and precision under changing traffic. While most conventional and ML-based database models, as cited in [15,19], depend on centralized architectures and are optimized for average or stationary load patterns, our framework uses Split Learning to enable real-time, distributed decision making without sacrificing efficiency or scalability.

## 5. Conclusions and Future Work

This paper introduced a novel Collaborative Split Learning-based Dynamic Bandwidth Allocation (CSL-DBA) framework designed to meet the evolving demands of next-generation 6G-grade TDM-PON networks. By decentralizing the learning process and enabling ONUs to contribute to bandwidth prediction without sharing raw data, CSL-DBA achieves the right balance between privacy preservation, scalability, and real-time adaptability. Through extensive simulations under low, fluctuating, and high traffic conditions, the proposed model consistently demonstrated high accuracy (>99.6%) while maintaining low inference latency, even when the number of ONUs scaled from 8 to 64. In contrast to traditional rule-based, centralized, or QoS-driven DBA schemes, CSL-DBA demonstrated superior performance in handling bursty traffic patterns, reducing latency overhead and enhancing responsiveness under network congestion. Comparative analysis with current state-of-the-art DBA approaches confirmed that CSL-DBA meets the requirements for dynamic and intelligent bandwidth management in next-generation optical access networks. Its distributed architecture, built-in learning intelligence, and compatibility with emerging MEC and fronthaul paradigms make it a future-proof solution for 6G deployment scenarios. Future work will explore model compression, latency-aware training strategies, and integration with network slicing and cross-layer optimization techniques to further enhance real-time performance and deployment feasibility in large-scale optical access infrastructures.

## Figures and Tables

**Table 1:** Benchmarking the proposed CSL-DBA against existing DBA solutions.

DBA Solutions	Reference	Traffic Adaptability	Accuracy	6G Readiness
CSL-based	This Work	High: real-time adaptability to variable loads	High: >99.6% under diverse conditions	Yes: engineered for 6G optical edge
DL-based	[15,17,19]	Moderate: effective with trained models only under static conditions	High: 97∼99% under fixed traffic loads	Partial: supports 5G scenarios but lacks 6G optimization
Security-Enhanced	[26,27]	Low: rule-based thresholding with limited dynamic response	Moderate: dependent on attack detection efficiency	No: static logic, lacks predictive capability
QoS-Aware	[9,11,21]	Low: based on pre-established static policies	Low: inflexible to sporadic or real-time traffic	No: legacy network compatibility only
SL-based	[20]	Low: not assessed specifically for PON traffic	High: demonstrated in alternative sectors (healthcare, IoT)	Yes: fundamental framework for 6G learning paradigms
Energy-Aware	[7,12]	Moderate: indirect adaptation via energy metrics	Moderate: accurate under balanced load scenarios	Partial: energy-focused, lacks AI-driven flexibility
Fronthaul-Aware	[1,8,10]	Moderate: optimized resource deployment for edge computing	Variable: dependent upon network orchestration granularity	Yes: suitable for scalable 5G/6G deployments

## Data Availability

Data is contained within the article.
